# Primary and secondary patient participation - a qualitative study of healthcare professionals’ perceptions and experiences

**DOI:** 10.1186/s12913-026-15039-3

**Published:** 2026-06-27

**Authors:** Therese Scott Duncan, Carolina Wannheden, Maria Hägglund, Sabine Koch, Sara Riggare

**Affiliations:** 1 Participatory eHealth and Health Data (PATH), Department of Women’s and Children’s Health, Uppsala Universitet, Uppsala, Sweden; 2 Medical Management Centre, Department of Learning, Informatics, Management, & Ethics (LIME), Karolinska Institutet, Stockholm, Sweden; 3 Uppsala University Hospital, Uppsala, Sweden; 4 Health Informatics Centre, Department of Learning, Informatics, Management, & Ethics (LIME), Karolinska Institutet, Stockholm, Sweden

**Keywords:** Patient participation, Healthcare professionals, Informal caregivers, Digital solutions, Qualitative study

## Abstract

**Background:**

Patient participation is increasingly emphasized in healthcare policy and practice, still less is known about how healthcare professionals (HCPs) perceive and experience patient and informal caregiver participation. Patient participation as a concept is described as sharing something in a partnership, such as a shared decision. Meaningful participation requires bridging the knowledge and power gap between HCPs and patients. The aim of this study was therefore to contribute to the conceptualization of patient and informal caregiver participation based on HCPs’ experiences and perceptions.

**Method:**

We conducted an inductive thematic analysis of qualitative data from open-ended responses and comments in a national web survey in Sweden. The survey explored attitudes toward patient participation and workplace support for collaborative practices and was completed by 279 participants, whereof 247 (89%) responded to at least one open-ended question or free text comment.

**Results:**

Two overarching themes were generated. *Primary participation* refers to how patients and informal caregivers manage their own health at a micro level in society. *Secondary participation* refers to an organizational level in society, to influence the healthcare system and support peers at macro and meso levels. HCPs valued well-informed patients as partners but expressed concerns about misinformation, workload, and health inequities. Digital tools were seen as enablers of participation, though usability and organizational readiness posed challenges. Secondary participation was perceived as beneficial but hindered by structural barriers, lack of management support, and limited resources.

**Conclusion:**

HCPs generally supported patient participation, however, they faced systemic and practical obstacles. To foster meaningful collaboration, healthcare organizations need structured forums, training, and strategies to integrate patient knowledge at both individual and organizational levels. Future research should explore interventions that strengthen shared learning for patients and informal caregivers.

**Supplementary Information:**

The online version contains supplementary material available at 10.1186/s12913-026-15039-3.

## Background

Health systems worldwide emphasize patient participation in healthcare and urge a shift from traditionally paternalistic models to more collaborative ones [1, 2]. To engage patients as active partners, in decisions about their own care and to shape health services, is seen as key to improving care quality, patient satisfaction, and health outcomes [3]. However, meaningful participation requires bridging the knowledge and power gap between healthcare professionals (HCPs) and patients [4].

### Involvement, engagement and participation

The semantic difference between involvement, participation, and engagement relates to the role of the patient. Involvement is the broadest concept of these three and the least formal term, referring to a minimum amount of interaction expected from patients to be involved in their care and self-care. Engagement comes with a notion of a promise to be invited to engage, and participation as a concept has been described as to share something in a partnership [5], such as a shared decision, and will be the concept used in this study. Shared decision-making and patient participation can be influenced by HCPs, potentially influencing patients to align with the needs of the healthcare system [3]. Shared-decision making is usually based on a HCP-patient relationship where HCPs are considered as experts on medical evidence and patients regarded as experts in their personal health and everyday life [6]. To be involved in one’s care does not always require a patient-HCP relationship, since the digitalization of society has increased the possibility to be engaged in self-care and connect with peers.

### Roles of patients, informal caregivers, and healthcare professionals

The advancement of online patient platforms and communities empowers patients and informal caregivers to participate more. This leads to an integration of patients’ knowledge into the healthcare context, and a role shift where patients and informal caregivers become active contributors to healthcare [7]. More active and empowered patients and informal caregivers may affect the patient-HCP relationship [8]. When HCPs are open to accept knowledge gained outside the healthcare context, it could lead to an exchange of expertise [9]. Further factors affecting levels of patient participation are HCP’s believes, attitudes, and type of healthcare setting, complexity of tasks, patient-related demographic factors and severity of illness can impact the ability and willingness to participate [10].

Seibaek [11] states that patient participation is important for knowledge and skill development for HCPs, as well as for patients and informal caregivers. Hence healthcare systems need to facilitate increased patient participation [11]. The academic literature describes how HCPs can cultivate patient participation [6] and increase mutual engagement [12]. However, there is limited research on how patient participation affects HCPs and healthcare organizations’ efforts to meet increased demands of patient participation. Against this backdrop, we conducted a national survey in Sweden (*n* = 279) to explore HCPs’ attitudes and experiences towards working with patient participation, as well as their perception of relevant workplace support [13]. Quantitative findings of this survey showed that respondents generally viewed patient participation positively and some had learned new knowledge or skills from engaging with patients, but not many reported that such collaborations were fully supported by their workplace. The survey also highlighted HCPs’ concerns that greater patient participation may lead to increased workload or health inequities, if only a specific group of patients would actively participate and receive better care [13, 14]. These patient lead users (referred to as *spetspatienter* in Swedish) are patients or informal caregivers who take more responsibility for managing their health and meet their health-related challenges in a constructive, knowledge-based way, often also using their knowledge and expertise to improve healthcare for themselves and others [13]. However, more knowledge regarding patient participation in different contexts is needed.

Therefore, the aim of this study was to contribute to the conceptualization of patient and informal caregiver participation based on healthcare professionals’ experiences and perceptions.

## Method

We used a qualitative approach to analyze responses from open-ended questions and comments of an open web survey disseminated to HCPs in Sweden. This web survey and its quantitative findings have been described in detail by Duncan et al. [13].

## Study design

The Checklist for Reporting Results of Internet E-Surveys (CHERRIES) [15] was used (see Additional file 1). The survey was voluntary to answer and responses were anonymous. It consisted of 64 closed questions required to answer, covering demographics and 10 sections relating to different topics representing different empowering behaviors of patient lead users, as being described in Duncan et al. [14]. Within each section, participants responded to closed questions and had the option to add free text comments relating to the topic (Table 1). There were four additional open-ended questions to elicit further details. All free text comments and open-ended response questions were non-mandatory to answer. In the present study we focused on the rich qualitative comments provided by the respondents. The full survey is presented in Additional file 2.

**Table 1:** Description of sections, free text comments, and open-ended response questions

Sections	Open-ended questions and comments (number of responses)
Awareness of the term “spetspatient”	Comment (*n* = 24)
Knowledgeable patients and informal caregivers	Comment (*n* = 60)
To learn from patients and informal caregivers	Comment (*n* = 52)
Need for alternative ways to interact with healthcare	Comment (*n* = 97)
To coordinate healthcare contacts between different healthcare units	Comment (*n* = 65)
Patients perform self-tracking on their own initiative	Comment (*n* = 60)
Use of digital solutions to manage health conditions	Comment (*n* = 35) What negative aspects do you perceive when digital solutions to manage health conditions are used? (*n* = 198)
Innovations by patients and informal caregivers	Comment (*n* = 50)
Patients and informal caregivers communicate their experiences	Comment (*n* = 68)
Patients’ and informal caregivers’ participation in healthcare unit development	Comment (*n* = 44)
Separate section for open-ended questions	What do you consider is the best with your workplace in regard to how you collaborate with patients and informal caregivers? (*n* = 208)
	What challenges do you perceive when patients and informal caregivers wish to be more engaged? (*n* = 211)
	What support exists within your workplace for existing challenges? (*n* = 178)

### Data collection

The survey was distributed using an exploratory, non-probability sampling strategy, with convenience sampling as a technique [16]. Inclusion criteria for the respondents were being HCPs (physician, nurse, other health professions with license, and non-licensed health professionals), Swedish-speaking, and age > = 18 years. Distribution of the survey was conducted in two rounds, with a first round of distribution using convenience sampling in purposefully selected healthcare units. The selection of healthcare units was based on five patient-driven innovations studied by the research program that funded the previous survey paper [13]. The operational managers at the five specialist healthcare centers (representing oncology, diabetes, neurology, psychiatry, and rheumatology) distributed the survey link to their staff by e-mail. In three of these healthcare centers, patient councils had been established to foster engagement and participation. The second round of distribution was done through an open advertisement by a medical university in Sweden, with no initial contact with potential respondents. The advertisement targeted HCPs nationally in different healthcare settings, and was disseminated through social media (Facebook). No incentives were offered to the participants.

Microsoft Teams was used for collecting data and the survey dissemination was performed between April 2021 and February 2022, by distributing a link to a web page describing the survey and participant information. From the web page it was possible to use the link to the survey. Due to the ongoing pandemic, the data collection period was extended from the original plan. The survey was pilot tested with six cognitive interviews and 30 respondents to the survey. The survey was completed by 279 participants, whereof 247 (89%) responded to at least one open-ended question or comment. The first round of distribution led to a response rate of 18% (*n* = 86/478 distributed surveys), and the second round resulted in a response rate of 36% (*n* = 193/536 hits on the link to the survey). The survey respondents in distribution one answered the comments and open-ended questions to a greater extent (90% compared to 84% in distribution two).

### Data analysis

All collected qualitative data were analyzed using an inductive thematic analysis (TA) approach [17]. An inductive approach was chosen to enable themes to develop from the data without imposing pre-conceived categories, thus capturing participants’ perspectives in an unbiased, data-driven manner (16). The analysis was conducted in six stages: (1) familiarization of the collected data by reading it through and selecting quotations by all authors, (2) categorization of data into meaning units with keywords by TSD and SR, (3) coding of all data into two themes by TSD and SR, (4) naming of themes to appropriately fit the aim of the study by SR 5), conceptualizing through interpretation of the keywords, codes and themes by TSD, and 6) presenting the final result by all authors.

## Results

**Fig. 1:**
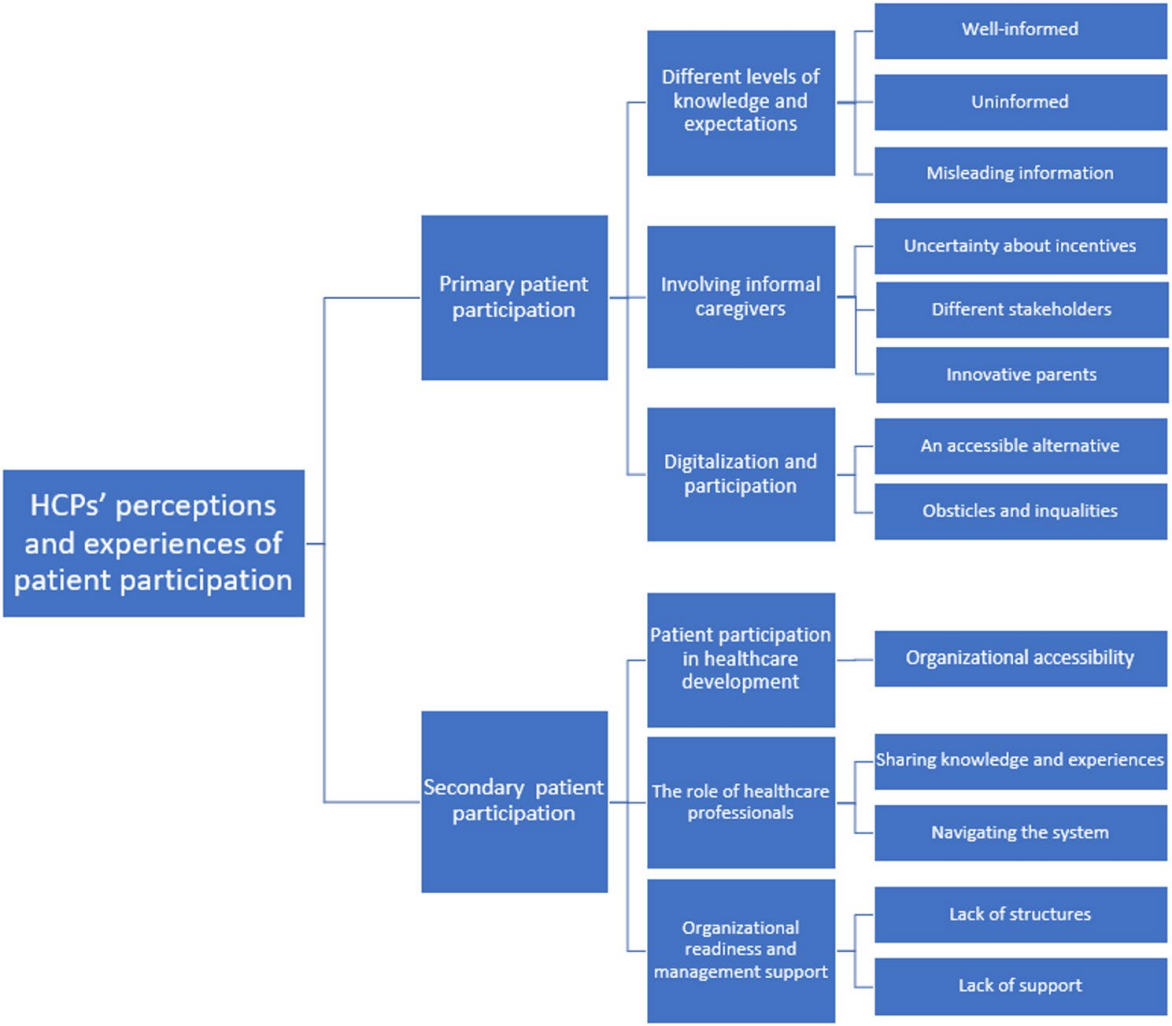
Themes and sub-themes from the TA

During the analysis process, it became apparent that healthcare professionals’ experiences of patient and informal caregiver participation differed depending on what type of participation they referred to. We therefore propose two main themes, distinguishing primary and secondary patient participation, each composed of three sub-themes (Fig. 1). Primary participation refers to patient participation to manage their own health at a micro level in society, whereas secondary participation refers to patient participation on an organizational level in society, to influence the healthcare system and support their peers at macro and meso levels.

Table 2 presents the demographics and professional characteristics of the 247 respondents who provided free text comments or answered open-ended questions. All regions of Sweden are represented in the sample. Secondary healthcare (71%), females (90%), and nurses (48%) are over represented. Almost all participants had worked more than one year in healthcare (95%).

**Table 2:** Participant characteristics

Age *n* (%)	Gender *n* (%)	Workplace *n* (%) (multiple answers)	Profession *n* (%)	Time worked within healthcare *n* (%)
19–29 24 (10)	Male 22 (9)	Primary healthcare 64 (26)	Physician 39 (16)	< 3 months 2 (1)
30–39 58 (23)	Female 223 (90)	Secondary healthcare 176 (71)	Nurse 119 (48)	3–12 moths 11 (4)
40–49 77 (31)	Prefer not to say 2(1)	Digital healthcare 3 (1)	Other health profession with license 61 (25)	> 12 months 234 (95)
50–59 63 (26)		Other 23 (9)	Non-licensed health profession 28 (11)	
60–69 23 (9)				
> 70 2(1)				

### Primary patient participation

Primary patient participation encompassed HCPs’ experiences of patient and informal caregiver participation at an individual care level. This theme included sub-themes related to meeting patients and informal caregivers with different levels of knowledge and expectations, involvement of informal caregivers, and the connection between digitalization in healthcare and self-care in regard to patient participation.

### Different levels of knowledge and expectations

In their work, HCPs need to involve patients and informal caregivers whose level of knowledge may vary considerably. Individuals can range from well-informed to mis-, and uninformed. Overall, it was considered less complicated to engage well-informed patients and informal caregivers. The vast availability of misleading information was mentioned as a concern by the participants, with the potential of harmful situations. HCPs reported that patients vary widely in their health knowledge and preparedness, which influences how easily they can be involved.

*My own perception is that about a quarter of the patients I meet are well-informed, actively seek information, and ask for the ‘latest standard of care’.* (Physician, woman, secondary care)

When patients were well-informed about their condition, HCPs viewed them more as collaborators and could individualize care based on mutual knowledge. HCPs and well-informed patients were generally regarded as collaborators in managing health conditions, although the HCPs experienced that the degree of patient autonomy varied. Some patients took a more active role in overseeing their condition and coordinating healthcare interactions.

When a treatment process proves ineffective, some respondents had observed that well-informed patients tended to become more assertive in their healthcare interactions. This also occurred when patients sought greater participation in their care than the healthcare system was able to provide. Additionally, the participants considered that some patients felt compelled to be knowledgeable due to their low expectations about HCPs’ expertise. However, when patients acquired accurate and relevant information they were perceived as well-prepared, advocating for the latest standards of care. HCPs considered they could place greater trust in well-informed patients’ competence and knowledge, allowing for a more individualized approach to treatment.

*Informed patients work WITH me so we can find the best solution. They also bring a humble, open, and curious approach to the process.* (Physician, woman, secondary care)

The participants described that one way for patients to increase their knowledge and understanding of health and medication regimes, was to self-track, which can be done with or without the use of technology. The HCPs believed systematic monitoring of medication side effects was beneficial for patients, when medication regimes were changed. It was described as particularly constructive when patients documented early symptoms and tracked their mood and overall well-being over time. By systematically monitoring such health-related data, individuals could gain a deeper understanding of their own condition. A potential negative aspect of self-tracking was also raised, namely the risk of focusing excessively on negative disease-related aspects of living with a chronic condition.

*That they want to track themselves means that they are participating in their own care and present in what is happening, what decisions are being made, and which treatment options they can choose from. It means that I, as a nurse, must use my knowledge in a different way to provide care.* (Nurse, woman, municipality care)

Some respondents described that their patients did not have the capability to share or even discuss their disease knowledge. Older patients were frequently perceived to lack capability and knowledge. Respondents working in emergency care believed that patients with more acute disabilities and conditions are often not knowledgeable, and that HCPs in such acute care contexts do not have the same collaboration with their patients as in other parts of the healthcare organization.

Respondents expressed concern that being uninformed could lead to health and care inequalities. However, despite believing patients to have insufficient knowledge, some of the respondents were reluctant towards patients that learned more about their condition outside healthcare. It was considered subjective and could therefore not be reliable, and in addition not available for everyone, which might increase health inequity.

*Provided that the patient relies on trustworthy sources. I often encounter patients who have turned to unreliable groups on social media and searched questionable websites. This is particularly common among parents of children who, for example, demand tongue-tie release for a non-existent posterior tongue-tie, or suspect that factors such as diet are the cause of their child’s severe medical condition. I experience that well-informed patients are rarely critical of their sources. (*Nurse, woman, primary care)

The respondents emphasized the importance of being perceptive and ensuring a clear understanding of what the patient has derived from health-related information. Since treatment solutions vary between individuals, discussions must be approached with sensitivity to avoid escalating into a power conflict. While discussions about the condition itself were encouraged, treatment options may require more careful handling to prevent misunderstandings and unrealistic expectations.

*It can be a challenge when a patient has done a lot of reading but hasn’t fully understood what they’ve read. In such cases, it’s important to be attentive to this and try to grasp what the patient has understood—or believes they know.* (Physician, woman, primary care)

Given the vast availability of information, some of which may be misleading or harmful, respondents considered it crucial to guide patients with limited medical knowledge toward reliable sources. Respondents noted that patients that were more exposed to inaccurate information sometimes requested treatments that were unsuitable or potentially dangerous. Challenges were also mentioned when patients found relevant information but misinterpreted critical aspects of it. When patients challenge HCPs’ knowledge, particularly in cases where HCPs may lack the necessary expertise to offer adequate support, the participants considered it difficult. Collaboration with such patients and informal caregivers was perceived as highly demanding, especially when patient participation shifted into excessively demanding or threatening interactions, or where expectations did not align with the support the HCPs could provide due to legal and regulatory limitations. The respondents found it increasingly difficult to navigate these situations effectively and move forward.

*It is not positive when patients consider themselves the only experts in the room or that my humble and open attitude is the same thing as ignorance or lack of competence.* (Physician, woman, secondary care)

*Codependent and overly involved patients and informal caregivers who do not listen or believe us as healthcare professionals. Some think they know better than physicians and nurses, but clearly lack understanding and knowledge. At the same time, these patients and informal caregivers live their reality and know their own bodies, or their loved one.* (Nurse, woman, secondary care)

### Involving informal caregivers

Involving informal caregivers was seen to support the patient’s participation (especially when the patient’s capacity is limited) and reduce patient burden. They were considered as a vital support to patients (e.g. in treatment management and adherence), as well as an important informational resource for healthcare staff. However, the respondents experienced that the healthcare system was not built to meet the needs of informal caregivers, and it was therefore more challenging to systematically involve them as partners in consultations about a patient’s care.

Uncertainty about informal caregivers’ incentives was another challenge that respondents raised when collaborating with informal caregivers. Some respondents described informal caregivers as occasionally acting out of desperation, sometimes even displaying manipulative behavior.

*I appreciate it when informal caregivers are well-informed and knowledgeable, but within my professional field, that knowledge can sometimes be misapplied. In my experience, it can also lead to a kind of learned helplessness or exploitation of the system. We haven’t actively worked with family members, even though we recognize that the need is significant*. (Behaviorist, woman, mental care)

Additionally, respondents noted that informal caregivers often wished to discuss a patient’s condition with HCPs. However, confidentiality regulations make it difficult to accommodate such requests and meet informal caregivers on these terms. If a patient explicitly refused to allow HCPs to communicate with an informal caregiver, this decision was respected. Instead, the HCPs believed it was positive for patients and informal caregivers to discuss with their peers and recommended patient associations for them to share their experiences. However, the HCPs stressed the importance of seeing patients and informal caregivers as different stakeholders to ensure that informal caregivers do not take over the conversation, while at the same time making sure that the voices of informal caregivers are not excluded.

*Patients and informal caregivers have different views on the level of care and treatment.* (Nurse, woman, palliative care)

Parents serving as informal caregivers were widely regarded as experts on their children’s needs and well-being. They provided valuable insights to HCPs on effective strategies or interventions, such as practical assistive devices, to enhance their children’s daily lives and overall well-being.

*I don’t have the knowledge of how the illness affects the patient. If the patient or their relative knows how the illness/treatment impacts their everyday life, that is information I, as a nurse, need to be professional and provide the best possible care. Furthermore, it enhances patient safety by minimizing the risk that I might miss something important to the patient.* (Nurse, woman, municipality care)

### Digitalization and participation – enabler or obstacle?

When discussing digital tools used by patients or informal caregivers, the HCPs mentioned tools to monitor a condition, to read the medical record online, to have online encounters, and to use the Internet to find information and support groups. Overall, the respondents regarded digital tools as valuable complements, or even primary tools, for managing various health conditions. These solutions were experienced to contribute to time and energy efficiency for both healthcare providers and patients and to serve as a motivating factor for patients in their self-care practices.

*Saves time and energy for both healthcare professionals and for patients and their informal caregivers.* (Speech therapist, woman, secondary care)

One example was to use digital tools for continuous measurement of physiological parameters, such as blood pressure and the identification of patterns over time. This practice was viewed positively since it did not require advanced medical expertise and was undertaken as part of self-care. However, it could also place new demands on the HCPs to adapt their work processes.

The respondents acknowledged that the expansion of digital communication technologies has enabled patients and informal caregivers to engage with healthcare providers in various ways, including remote interactions. Given that it could sometimes be challenging for patients to participate in physical clinical encounters, the respondents believed digital solutions for communication were often preferred as a more accessible alternative, which could facilitate continued participation. They also described that digital solutions resulted in shorter interactions with the patients.

However, some barriers to overcome regarding digital systems supporting patient-provider interaction were acknowledged. Not all clinical interactions can be replaced with digital consultations, despite the rapid shift toward remote healthcare solutions during the pandemic. Digital encounters were particularly suitable for follow-up appointments, and digital tools served as effective means for disseminating information. The participants described that telephone communication remains common, as some HCPs lack sufficient familiarity with digital platforms and technologies, as well as that the current state of digitalization for patient participation in healthcare was not user-friendly. Several respondents considered their workplaces unsuitable for online consultations, particularly in cases where physical examinations were essential, such as in elderly care, palliative care, mental health care, emergency departments, and in prehospital emergency medical care. Online encounters as solutions for communication were proposed as being more applicable to primary healthcare, although some primary healthcare HCPs disagreed.

*I find that platforms like Zoom and similar do not provide the same connection as physical meetings. The clinic would probably collapse if there were more contact channels (physical meetings, phone contact/advice, My Healthcare Contacts/1177, email, and digital meetings).* (Midwife, woman, primary care)

*Usability challenges… Some do not manage it, leading to decreased motivation.* (Physician, man, secondary care)

Digital systems must function effectively and be easy to use, yet their practical applications were often difficult to comprehend. The fact that parts of the electronic health record are made available to patients in Sweden also led to some concerns. Respondents noted that the language used in online medical documentation is highly medical and not patient-friendly, frequently leading to misunderstandings. There were also concerns about patients reading their electronic health record online before being given information from their physician (e.g., diagnoses or lab results). This was considered leading to increased anxiety and unanswered questions that could lead to misunderstandings. HCPs generally did not consider social media platforms and online peer-to-peer forums to be optimal and therefore did not recommend them, due to the risk of increased anxiety and concerns when not being structured by an HCP.

*It can be difficult for the patient and their informal caregivers to navigate… They receive incorrect information.* (Nurse, woman, medical care at home)

E-mail communication was considered an insecure method for patient interactions, and not all patients feel comfortable using digital solutions, according to the respondents. Additionally, lack of available equipment for technical integration further complicates things. Integrating digital tools into healthcare remains a complex and lengthy process according to the participants, as such technologies are classified as medical devices and must meet evidence-based requirements. Additionally, respondents indicated that innovative and creative approaches from HCPs were not widely encouraged by healthcare organizations or the management.

Inequalities were also mentioned as a challenge when using digital tools, since not all patients have the ability to use the technology. The respondents were concerned that lack of abilities could lead to inferior health care for these patients and less knowledge about their condition.

*There is a risk that individuals who are unable to manage or access technology will, in the long run, suffer from informational injustice and inequality, which may lead to poorer healthcare for the individual*. (Nurse, woman, secondary care)

### Secondary patient participation

Secondary patient participation refers to patients’ and informal caregivers’ participation at the organizational level of healthcare (e.g., care coordination), as well as contribution to improvements in healthcare services and policies beyond their own direct medical care (for example by sharing experiences, innovations, and peer support). This theme included sub-themes related to patient and informal caregiver participation in healthcare development, what is the role of HCPs within secondary participation, and organizational conditions and support.

### Patient and informal caregiver participation in healthcare development

Some of the respondents described a welcoming environment that encourages patient engagement, emphasized the importance of eliciting patients’ knowledge by fostering open communication, learning from patients’ experiences, and collaborating with them in a flexible manner. Organizational accessibility was regarded as an essential factor in facilitating patient engagement, along with teamwork and open dialogue to address the needs of both patients and informal caregivers. To exemplify how patient participation was sought and supported, some respondents described how their healthcare units welcomed suggestions through various channels, including suggestion boxes, investigative reviews, follow-up surveys, interviews, and evaluations. These inputs were systematically mapped to assess feasibility and implement selected recommendations.

*After all, it’s for them we exist, and we believe our encounters and treatment processes become healthier and more humane—not ‘us’ and ‘them’. We strive for open collaboration, for example by formulating goals, identifying what matters in life, and ensuring that we’re working on what is truly important to them.* (Counselor, woman, primary care)

Still, some participants considered it to be too difficult for an external person, such as a patient or informal caregiver, to understand the organizational prerequisites, and therefore participate in healthcare development.

### The role of healthcare professionals in secondary participation

The respondents saw an opportunity for them to facilitate secondary participation by encouraging patients and informal caregivers to share successes, positive experiences, as well as self-developed innovations for self-care with peers. In addition to patient experiences, patient-developed innovations were considered valuable to foster open dialogue. Respondents were positive to share validated patient-developed solutions with other patients. However, few had encountered such innovations and emphasized the importance of evidence and validation to ensure that no risks are introduced. To share lived experience and knowledge was believed to help both the person sharing their experiences (for example by contributing to decrease the loneliness of having a chronic condition) as well as peers (for example by receiving comfort, guidance, and even experience shared joy).

*I have experienced positive situations where patients have been able to share their success stories, which in turn have helped comfort or reassure fellow patients. (*Nurse, woman, secondary care)

However, the participants considered their role as a guardian, not to recommend patients or informal caregivers to share negative experiences with peers, as well as not to compare treatments, examinations, and medications.

Several of our respondents shared knowledge and best practices they had acquired from patients or informal caregivers with their closest colleagues as well as other patients. Fostering sustainable participation of patients and informal caregivers within the healthcare organization was reported as a learning experience for the HCPs. Overall, respondents suggested that opportunities for systematic patient participation in improvement work and collaborative learning are still limited.

*… That’s probably the kind of thing I, as a clinician, add to my own bank of experience—something I do share with other patients. But there are few structured opportunities within the team to reflect on and exchange what patients or their relatives have said, heard, experienced, or read.* (Occupational therapist, woman, habilitation center)

While they encouraged active participation of patients and informal caregivers, respondents emphasized that HCPs need to be mindful of the additional workload on patients and informal caregivers who need support and relief. For example, participation in care coordination can sometimes become a significant burden and challenge for patients and informal caregivers. Care coordination is particularly challenging when various contacts and levels of care need to be coordinated (for example social services, primary care, home care, psychiatry, and specialist healthcare), which can lead to frustration and anxiety. Considering that patients and informal caregivers may not always have the knowledge necessary to determine which contacts that need to be established, respondents suggested that HCPs may need to be more involved and take responsibility for care coordination, thus supporting patients in navigating the healthcare system.

*While it can be positive for patients to have agency and actively coordinate their care contacts, we cannot assume that all patients have that ability. Some may need support, and in certain cases, it may be beneficial for healthcare staff to be involved in the coordination.* (Counselor, woman, secondary care)

### Organizational readiness and management support for secondary patient participation

Lack of structured forums for secondary patient participation and undervaluing patient knowledge within the organizations were identified as barriers. The HCPs also lacked management support when difficult situations occurred. Nevertheless, the participants strived to tailor care to align with patients’ individual needs, and shared their acquired knowledge with their colleagues.

Although respondents from various organizations described successful examples of patient participation in healthcare development, many also highlighted lack of resources and time necessary to effectively implement such engagement in practice. Most respondents were either unaware of existing organizational support or explicitly stated that no such support was available when facing demanding situations with secondary patient participation.

*The doctors can support each other and, to some extent, other professional groups. There is no support from management. It’s not something that is discussed at the group level.* (Non-licensed health professional, woman, secondary care)

Further it was described that it can be a challenge to find a common ground with patients and informal caregivers who would like to participate at an organizational level, since they may not be sufficiently knowledgeable about organizational aspects or limitations.

The respondents emphasized the need for a healthcare system that automatically facilitates collaboration between patients or informal caregivers, ensuring meaningful engagement. Such a system could support patient-driven innovations, provide a platform for discussing patients’ prior knowledge, and to increase patient participation in healthcare development. The HCPs observed that patients are willing to engage and contribute more than current structures allow. However, the participants described that implementation of such participation is hindered by lengthy decision-making processes, outdated attitudes among healthcare providers, hierarchical structures, and the difficulty of initiating change within regional healthcare systems.

*There are lengthy decision-making processes within the regions and a strong hierarchical structure within the hospitals. So far away from personal-centered care, due to time constraints and that health consultations with healthy individuals occur at the expense of people with chronic conditions. There is a need for continuity, accessibility, and personal- centered care.* (Nurse, woman, secondary care)

Healthcare regulations could also pose challenges, as patient-driven innovations or solutions cannot be implemented or recommended unless formally approved. Instead, the participants primarily relied on colleagues for assistance, with support occurring on a more personal level. These challenges often stem from time constraints or limited resource capacity. Some HCPs perceived a lack of interest in their work, as well as insufficient understanding from management in regard to their responsibilities. Additionally, the absence of clear task descriptions further complicates their ability to navigate such situations effectively.

*That the design and organization of healthcare tends to hinder rather than enable approaches and structures—ones the patient is expected to fit into, rather than be adapted to.* (Nurse, woman, secondary care)

*Increased patient empowerment is currently in focus, not least politically, which makes it difficult to engage in a constructive discussion about the challenges.* (Physician, woman, secondary care)

## Discussion

Two themes describe how patient participation is conceptualized based on HCPs’ experiences and perceptions. The first theme, primary patient participation, centers on patients managing their own health, either through self-care or in interactions with HCPs. Primary patient participation is typically at a micro level and in alignment with person-centered care principles. The second theme, secondary patient participation, reflects how patients’ and informal caregivers’ participation extend beyond individual care by also engaging in navigating the complex healthcare system, supporting peers, and participating in quality improvement initiatives or governance at organizational levels.

### When knowledge is being challenged

The result of the study describes complex power dynamics between HCPs, patients, and informal caregivers when knowledge is being challenged. In the current study as well as in the literature a central concern is the widespread availability of misleading health information, which can compromise patient safety and complicate clinical decision-making [18, 19]. It is also considered important to ask patients about their online searches and guide them [19]. Still, in a previous publication by Duncan et al. [13] as well as in the current study, HCPs appreciated the value of knowledgeable and innovative patients. The previous publication described that 96% of respondents had positive attitudes towards patients described as knowledgeable about their condition and they viewed patient engagement in developing clinical services as beneficial in theory [13]. Further, the van Riel et al. describe HCPs consider positive effects on consultation when patients have used online search and information [20], as well as for treatment compliance and having an information-seeking coping style rather than an information-avoiding coping style (blunting) [21, 22]. Our results show that when patients acquired accurate and relevant information they were perceived as well-prepared, advocating for the latest standards of care with high health literacy and could be engaged as partners in care.

Notably, the comments from the survey suggested some ambivalence: respondents cautioned that patient-acquired knowledge might also be misleading or lack context, potentially leading to unrealistic demands on care. Difficulties arose when patients challenged HCPs’ knowledge, that is described in the literature as a desire for HCPs to maintain control [13, 23] and non-compliant with patients lived experiences [24]. In our study, collaboration with such patients and informal caregivers was perceived as highly demanding, especially when patient participation shifted into excessively demanding or threatening interactions, or where expectations did not align with the support the HCPs could provide due to legal and regulatory limitations. The respondents found it increasingly difficult to navigate these situations effectively and move forward when the boundaries of professional roles and healthcare system capabilities were challenged. Overall, few HCPs had practical experience of collaborating with patients as partners. This also applies to involving informal caregivers. Caregiving by family members (informal caregiving) has a long-lasting tradition in self-care and as support to healthcare [25], and recently as an important role in health policy development [26]. Still, support for informal caregivers is insufficient [27] and healthcare systems are not meeting the informal caregivers’ individual needs [25]. This may result in HCPs, in the current study, showing limited enthusiasm toward informal caregivers, with concerns about informal caregivers’ motives and potential overreach. However, our findings indicate that parents of younger children are more readily accepted by HCPs, who demonstrate greater openness to the insights and knowledge these informal caregivers provide about their children.

JØrgensen et al. confirm that HCPs generally perceive patient participation as beneficial for better health outcomes [28]. In our study some HCPs viewed patient participation as a partnership, while others appeared to interpret it primarily as patients fulfilling their part to treatment adherence. Furthermore, the literature highlights that HCPs can consider patients’ and informal caregivers’ insufficient knowledge and constraints related to their health conditions as barriers to their participation in their health and care [28]. On the other hand, patients and informal caregivers with high health-literacy have reported being unsupported by the healthcare organization, and at times even obstructed, especially when their proactive behaviors do not align with healthcare’s expectations [14]. This can be seen as a form of epistemic injustice, where patients’ and informal caregivers’ knowledge is not acknowledged or valued [29]. While highly engaged patients and informal caregivers have expressed a desire to take on more active roles, such as being healthcare partners, innovators, and mentors, Duncan et al. describe that these patients and informal caregivers also wanted better support for tasks they were not equipped or interested in. Such as coordinating their own care, which was often imposed on them by the healthcare system [14].

### Barriers for patient and informal caregiver participation

The respondents of the current study identified several barriers to patient participation within their organizations, including lack of structured forums, undervaluing of patient knowledge, insufficient management support, and limited resources. Respondents called for a healthcare system that naturally supports meaningful collaboration, patient-driven innovation, and shared learning. When HCPs’ knowledge is challenged, it can hinder their willingness to promote greater patient and informal caregiver participation, as also being described in the literature [30]. Whereas the HCPs in the study, in general, considered well-informed patients as collaborators, they had limited experiences of working with empowered patients and informal caregivers having high health-literacy. They further found it increasingly challenging to meet patient demands, partly based on misinformation, and lacked workplace support in the area. According to the literature, HCPs also tend to exclude patients from decision-making when they perceive them as incapable of participating [31]. Rather than adapting their approach to involve patients and informal caregivers with varying levels of knowledge, HCPs may overlook opportunities to involve them in shared-decision making. The respondents in this study stated that patients or informal caregivers, as external individuals, do not fully understand organizational structures, which limits their ability to contribute to healthcare development. There were also concerns that not all patients have the ability to use for example technology to the extent needed for increased patient participation. Lack of abilities could lead to inferior collaborations with informational injustice and poorer health-literacy. Thus, better support structures for secondary patient participation are needed.

Additional barriers identified by participants in this study, as well as in existing literature, include time constraints caused by heavy workload [23]. Structural limitations are also reported in the literature, when evidence-based treatment plans can limit the ability to incorporate patient participation and needs of the patients and informal caregivers [28]. Other more institutional barriers can be lack of equipment, regulatory constraints, and limited support for innovations [28]. Absence of training in patient-caregiver relationships has also been described as one reason for lack of organizational readiness [23]. In this study, the institutional barriers were also seen as hindering the broader adoption of digital tools. The lack of ability to embrace creative approaches within healthcare organizations further underscores the need for systemic change to support digital transformation. The respondents generally viewed digital solutions as valuable complements, and in some cases, primary tools for managing health.

### Support to cultivate participation

Digital tools offer promising avenues for enhancing patient participation, such as allowing patients to view lab results, clinical notes, medication lists, care plans, and lowering the threshold for contact with HCPs, to increase understanding and encourage patients to be prepared and engage in discussions with HCPs [32]. However, effective implementation requires attention to usability, equity [32], and organizational readiness. Addressing these challenges is essential to ensure that digital health solutions contribute meaningfully to person-centered and inclusive care. To foster meaningful involvement in individual care as well as healthcare improvement at an organizational level, there is a need for support structures for both primary and secondary patient participation, to link patient knowledge to HCPs’ knowledge [33]. To further increase secondary participation, co-creation and methods with stakeholders could be used to increase organizational readiness [34].

The respondents in the current study saw value in encouraging patients and informal caregivers to share positive experiences and self-developed innovations, which could foster peer support and open dialogue. Sharing knowledge learned from patients and informal caregivers among colleagues and patients was common, but systematic opportunities for patient participation in improvement efforts remain limited. Previous literature on patient-driven innovations illustrates that patients and informal caregivers have developed and shared various innovations focusing on addressing unmet needs related to access to self-care support tools, open sharing of information and knowledge, and patient agency in self-care and healthcare decisions [35, 36]. While these innovations seem well aligned with the needs identified by the respondents in this study, there are also notable differences that would benefit from further investigation. For example, although respondents in this study emphasized the importance of peer-to-peer learning and sharing of information among patients and informal caregivers, they were reluctant to advise patients to use social media or digital forums. Concerns about safety and evidence were noted, where HCPs felt responsible for discouraging the sharing of negative experiences or treatment comparisons. The usability of digital systems was a concern, as well as patients’ online record access, which raised issues around language complexity and timing of information delivery, potentially leading to patient anxiety and misinterpretation. While these concerns were common among HCPs, the literature shows that patients report benefits from accessing their health records, including increased participation, improved preparation for medical encounters, a stronger sense of ownership, and improved knowledge of their condition [37–39], even though it can be discouraging when little context is provided or test scores are poor [39]. Alomar et al. state that the impact of patient access to EHRs increases treatment adherence, self-management, empowerment, HCPs communication and relationship, patient satisfaction, and better health outcomes [32]. The literature also indicates that access to online medical records for patients did not lead to increased anxiety [38, 40], although concerns have been raised among patients regarding misuse of data if available to third parties [39]. Overall, the participants in our study believed that patients’ and informal caregivers’ use of digital systems would lead to new demands to adapt workflows and interpret patient-generated data.

### Method discussion and future research

To ensure the anonymity of survey respondents, no registration or collection of personal data was required to answer the survey. IP-addresses were not tracked or stored. While this preserved the privacy of respondents, we cannot guarantee that no duplicate responses were submitted.

The design of the survey in Microsoft Teams did not allow the respondents to review their answers as a summary before submitting. Further, the survey questions and statements were designed to focus on positive aspects of patient participation, hence the answers could be affected by the positive tone in the statements. To adjust for this, a few open-ended questions were designed with focus on challenges related to patient and informal caregiver participation in general and negative aspects of the use of digital solutions in particular.

Since we used a non-probability sampling technique, the selection is based on availability. By using a non-randomized sample, with no equal representation, the result is not representative for the whole HCP-population in Sweden. The broad focus on health and inclusive inclusion criteria allowed for general perceptions across a range of healthcare contexts, however, this approach may also have contributed to less detailed insights into condition- or care specific practices. Further, female nurses were overrepresented among the participants, which reflects the female dominance for that occupation in Swedish healthcare. This may have influenced the findings as specific HCP may emphasize different aspects of clinical work, patient interaction, and organizational constraints.

To further investigate the concepts of primary and secondary participation, future research could focus on patients’ and informal caregivers’ perspectives, as well as social determinants of health that may influence participation.

## Conclusion

Our thematic analysis contributed to the understanding of different levels of patient participation and we identified challenges and needs to be addressed.

In general, many healthcare organizations are only starting their work on patient participation with the use of digital tools aiming for increased participation. Even though there are trust issues and a discussion concerning knowledge legitimacy regarding patients and informal caregivers, the participants considered that patients and informal caregivers can act as partners not only in managing their personal health but also in shaping and improving the healthcare system for the benefit of the wider patient community. HCPs tend to trust well-informed patients and high health-literacy allowed for a more individualized approach to treatment. The participants called for a healthcare system that supports shared learning, and a model for how to account for patients’ and informal caregivers’ knowledge, to meet the diversity of patients with different levels of health-literacy and technical understanding. Future research should further explore these issues, to explore interventions that strengthen shared learning for patients and HCPs.

## Supplementary Information

Below is the link to the electronic supplementary material.

Supplementary Material 1

## Data Availability

Anonymized data in Swedish is available by the first author.

## Abbreviations

HCP: Healthcare professional
TA: Thematic analysis
