# Supplementary material for: Primary and secondary patient participation - a qualitative study of healthcare professionals’ perceptions and experiences
Source: BMC Health Serv Res. 2026 Jun 27;26:876. doi: 10.1186/s12913-026-15039-3 (PMC13312649; doi:10.1186/s12913-026-15039-3)
Supplement: Supplementary file 1 — Supplementary Material 1 [file 12913_2026_15039_MOESM1_ESM.docx]

**Demography**  I consent to participate in the study “Patient Lead Users – a resource for healthcare?”

I consent to my personal data being processed in the manner described in the research participant information.

**Occupation**

**Region**

**Workplace/s**  Primary health care Specialized health care Digital health care Other

**Healthcare unit**

**For how long have you been working in health care?** < 3 months 3 – 12 months >12 months

**Age** 18-29 30-39 40-49 50-59 60-69 70>

**Gender** Woman Man Other option Prefer not to say

**10.** **Are you aware of the concept ”Patient Lead Users (Spetspatienter)”?** Yes No

**11. Comment**:

* = Mandatory

| ***12. Knowledgeable patients and informal caregivers*** |
| --- |
| **I believe it is beneficial for patients to be well-informed about their own condition. *** |
| **At my clinic, we assess patients’ prior knowledge regarding their condition**. * |
| **On several occasions, I have discussed patients’ knowledge about their condition with them. *** |
| **I believe it is beneficial for informal caregivers to be well-informed about the patient’s condition. *** |
| **At my clinic, we assess informal caregivers’ prior knowledge regarding the patient’s condition**. * |
| **On several occasions, I have discussed the informal caregiver’s knowledge about the patient’s condition with them. *** |
| **13. Comment:** |
| ***14. To learn from patients and informal caregivers*** |
| **I believe it is beneficial to acquire new knowledge or skills from patients. *** |
| **On several occasions, I have learned new knowledge or skills from patients. *** |
| **At my clinic, we regularly follow up what we learn from patients and informal caregivers. *** |
| **I believe it is beneficial to acquire new knowledge or skills from informal caregivers. *** |
| **On several occasions, I have learned new knowledge or skills from informal caregivers. *** |
| **15. Comment:** |
| ***16. Need for alternative ways to interact with healthcare*** |
| **I believe it is beneficial when patients wish to use alternative ways to communicate with me, rather than relying on in-person encounters. *** |
| **At my clinic, we follow up patients’ needs to use alternative ways to communicate with us. *** |
| **On several occasions, I have communicated with patients through alternative ways, rather than in-person encounters. *** |
| **17. Comment:** |
| ***18. Coordinating healthcare contacts between different healthcare units*** |
| **I believe it is beneficial when patients take the initiative to coordinate their healthcare contacts between different clinics. *** |
| **On several occasions, I have assisted in coordinating healthcare contacts between different clinics. *** |
| **I believe it is beneficial when informal caregivers coordinate the patient’s healthcare contacts between different clinics. *** |
| **19. Comment:** |
| ***20. Patients performing self-tracking on their own initiative*** |
| **I believe it is beneficial when patients independently self-track e.g. symptoms without a prescription. *** |
| **On several occasions, I have provided feedback to patients regarding their self-tracking. *** |
| **At my clinic, we encourage patients to independently self-track. *** |
| **21. Comment:** |
| ***22. Use of digital solutions*** ***(e.g. 1177, diagnosis-specific applications) to manage health conditions*** |
| **I believe it is beneficial when patients use digital solutions to manage their condition. *** |
| **At my clinic, we encourage the use of digital solutions. *** |
| **On several occasions, I have used digital solutions together with patients. *** |
| **I believe it is beneficial when informal caregivers use digital solutions to manage the patient’s condition. *** |
| **On several occasion, I have used digital solutions together with informal caregivers. *** |
| **23. What negative aspects do you perceive when digital solutions to manage health conditions are used?** |
| **24. Comment:** |
| ***25. Innovations by patients and informal caregivers*** |
| **I believe it is beneficial when patients create their own innovations for managing their condition (innovations = new for the patient group, simplify daily life, and contribute to increased perceived health). *** |
| **I believe it is beneficial when informal caregivers create innovations for the patient’s condition. *** |
| **My clinic supports me to work with patient or informal caregiver innovations. *** |
| **On several occasions, I have encountered patients’ innovative solutions. *** |
| **On several occasions, I have encountered informal caregivers’ innovative solutions. *** |
| **26. Comment:** |
| ***27. Patients and informal caregivers communicating their experiences*** |
| **I believe it is beneficial when patients share their experience with other patients. *** |
| **At my clinic, we encourage patients to share their experiences with other patients**. * |
| **On several occasions, I have encouraged patients to share their experiences with other patients. *** |
| **I believe it is beneficial when informal caregivers share their experience with other informal caregivers. *** |
| **At my clinic, we encourage informal caregivers to share their experiences with other informal caregivers**. * |
| **On several occasions, I have encouraged informal caregivers to share their experiences with other informal caregivers. *** |
| **28. Comment:** |
| ***29. Patients’ and informal caregivers’ participation in healthcare unit development*** |
| **I believe it is beneficial when patients engage in the improvement work of the clinic. *** |
| **At my clinic, patients have the opportunity to engage in the improvement work.** * |
| **On several occasions, I have encouraged patients to engage in the improvement work of the clinic. *** |
| **I believe it is beneficial when informal caregivers engage in the improvement work of the clinic. *** |
| **At my clinic, informal caregivers have the opportunity to engage in the improvement work.** * |
| **On several occasions, I have encouraged informal caregivers to engage in the improvement of the clinic. *** |
| **30. Comment:** |
| ***Open-ended response questions:*** |
| **31. What do you consider is the best with your workplace regarding how you collaborate with patients and informal caregivers?** |
| **32. What challenges do you perceive when patients and informal caregivers wish to be more engaged?** |
| **33. What support exists within your workplace for existing challenges?** |
